# Preferential use of unmutated immunoglobulin heavy variable region genes in Boxer dogs with chronic lymphocytic leukemia

**DOI:** 10.1371/journal.pone.0191205

**Published:** 2018-01-31

**Authors:** Emily D. Rout, Robert C. Burnett, Julia D. Labadie, Janna A. Yoshimoto, Anne C. Avery

**Affiliations:** Department of Microbiology, Immunology and Pathology, Colorado State University, Fort Collins, Colorado, United States of America; University of Kentucky, UNITED STATES

## Abstract

Human chronic lymphocytic leukemia (CLL) is a clinically heterogeneous disease, and immunoglobulin heavy variable region (IGHV) gene mutational status is an important prognostic marker. IGHV mutational status has not been previously examined in canine CLL. We sequenced the IGHV-D-J rearrangements from 55 canine patients with CLL, including 36 non-Boxer and 19 Boxer dogs. The majority of non-Boxers (75%) had mutated IGHV genes, whereas the majority of Boxers (79%) had unmutated IGHV genes. IGHV3-41 and IGHV3-67 gene usage was significantly higher in Boxers with CLL compared to non-Boxers. Additionally, 11 Boxers with large B-cell lymphoma and the normal IGHV repertoire of six control dogs (three Boxers and three non-Boxers) were sequenced. IGHV3-41 was preferentially used in Boxers with other forms of lymphoma and without lymphoproliferative disease. However, preferential use of unmutated IGHV genes was unique to Boxers with CLL, suggesting Boxers may be a valuable model to investigate unmutated CLL.

## Introduction

Human chronic lymphocytic leukemia (CLL) is the most common leukemia of adults in the Western world [1,2]. The disease has a variable clinical course, with wide ranges in time to progression and survival [3]. Analysis of the immunoglobulin genes has been crucial in understanding CLL pathogenesis and identifying subsets of patients with different clinical courses. Early studies identified restricted immunoglobulin heavy variable region (IGHV) gene usage in CLL compared to normal B-cells [4]. Later, studies demonstrated that the mutational status of the IGHV genes is highly prognostic and divides patients into subsets with different clinical outcomes [5,6]. Patients with mutated IGHV genes have a more favourable clinical course, while patients with unmutated IGHV genes have a poorer prognosis. Subsequently, subsets of unrelated CLL individuals were found to have highly similar to identical B-cell receptor immunoglobulins (stereotyped BCR) [7], which allowed for further stratification of patients and prognostication for certain subsets.

IGHV mutational status continues to be a major prognostic factor in human CLL [8] and more recently was shown to predict response to therapeutic agents [9]. The European Research Initiative on CLL has helped to establish standard methods for accurate analysis of mutational status [10,11]. Mutational status is determined by amplifying and sequencing the IGHV region, aligning the sequence to immunoglobulin databases, and calculating the percent identity between the case sequence and closest germline IGHV gene. Germline identity >98% is consistent with unmutated CLL, while cases with <98% identity constitute mutated CLL cases.

Canine B-cell chronic lymphocytic leukemia shares many features with human CLL. The disease is characterized by a clonal expansion of small B-cells in the peripheral blood. In people, the expanded B-cell population usually co-expresses CD5 and CD23 [12]. Canine CLL cells do not express the CD5 antigen and a CD23 antibody is not available in dogs. However, the clinical presentation and clinical course in dogs appear similar to that seen in human patients. The disease affects older dogs, with a median age at diagnosis ranging from 8–11 years [13–16]. Lymphadenopathy and splenomegaly are common, affecting approximately 50% of patients [13]. Cytologic review reveals the majority of lymphocytes are small with condensed chromatin and no apparent nucleoli, with fewer yet variable numbers of pro-lymphocytes. Anemia is relatively common, affecting 25–53% of patients across two studies, and thrombocytopenia and neutropenia are rare [13,14]. While it appears that many patients have indolent disease [14,17], one study [17] found a wide range in survival times (25 to >1000 days).

IGHV gene usage and mutational status have not previously been studied in canine CLL patients. Bao et al. [18] characterized the canine immunoglobulin heavy chain variable region, identifying 80 IGHV genes, 6 IGHD genes, and 3 IGHJ genes. These gene names have been modified to adhere to the conventions of the international ImMunoGeneTics (IMGT) information system (http://imgt.cines.fr, [19–21]), and the new names are used in this study (personal correspondence from M-P Lefranc; unreferenced). IGHV genes were classified into three subgroups, with 76/80 genes belonging to subgroup IGHV3 (previously VH1). Recently, Martin et al. have expanded the canine immunoglobulin locus annotation, describing 83 IGHV genes and 6 IGHJ genes [22]. Three studies have shown that canine IGHV-D-J rearrangements predominantly use IGHV3 subgroup genes [18,23,24], with IGHV3-38 (previously VH1-44) and IGHV3-19 (previously VH1-62) preferentially used in one study [18]. Heavy chain CDR3 length ranged from 7 to 17 amino acids (AA) in one study [18], and 5 to 27 AA in another [23]. IGHV gene usage and mutational status were investigated in canine diffuse large B-cell lymphoma [25,26], where IGHV3-38 was most frequently used.

We investigated IGHV gene usage and mutational status in a cohort of canine CLL patients, and compared the repertoire to patients with large B-cell lymphoma and to normal B-cells. We hypothesized that canine CLL patients would have a skewed IGHV gene repertoire and variable mutational status.

## Materials and methods

### Diagnostic criteria for CLL cases

CLL cases were selected from peripheral blood samples submitted to the Colorado State University Clinical Immunology (CSU-CI) laboratory for flow cytometric immunophenotyping. Flow cytometry was performed as previously described [27] and antibody combinations are listed in Table 1. CLL cases were defined as having >5,000 lymphocytes/μL, with a homogeneous expansion (>60%) of small lymphocytes expressing the B-cell marker CD21. Antibodies for CD19 and CD20 are not available in the dog but anti-CD21 reliably detects B-cells when combined with T-cell antibodies. Intracellular flow cytometry with CD79a and Pax5 antibodies is also available in the dog to detect B-cells, but these antibodies are not used in routine immunophenotyping in our laboratory. B-cell size was classified as ‘small’ when the ratio of the geometric mean of B-cell to neutrophil forward scatter (FS) was <0.60, which correlates to a B-cell FS value <400 U on our flow cytometer. In previous studies, dogs meeting these diagnostic criteria predominantly had an indolent clinical course [17] and clinical characteristics [13] similar to mutated human CLL.

**Table 1:** Antibody panels used for immunophenotyping.

Tube	Antibody specificity and fluorochrome
Panel 1 (two color)^a^	
1	None
2	M^c^ IgG1-FITC/CD45-PE
3	CD18-FITC/M IgG1-PE
4	CD4-FITC/CD8-PE
5	CD5-FITC/CD21-PE
6	CD3-FITC/CD45-PE
7	CD4-FITC/CD14-PE
8	Class II MHC-FITC/CD34-PE
Panel 2 (multicolor)^b^	
1	M IgG1-FITC/M IgG1-PE/M IgG1-Alexa 647/M IgG1-Alexa 700/M IgG1-PE-Alexa-750/M IgG1-Pacific Blue
2	CD3-FITC/CD25-PE/CD5-APC/CD8-Alexa 700/CD4-Pacific Blue
3	Class II MHC-FITC/CD22-PE/CD21-Alexa 647
4	Class II MHC-FITC/CD34-PE/CD5-APC/CD14-PE-Alexa 750
5	Class II MHC-FITC/CD18-PE/CD5-APC/CD14-PE-Alexa 750/CD4-Pacific Blue
6	CD5-FITC/CD45-PE/CD21-Alexa 647

^a^Panel 1 samples were analyzed using a single laser Coulter XL (Beckman Coulter, Inc, Brea, CA).

^b^Panel 2 samples were analyzed using a 3-laser Coulter Gallios (Beckman Coulter, Inc, Brea, CA).

^c^M, mouse.

Unless otherwise noted, all antibodies were purchased from Bio-Rad, Hercules, CA. Clones are as follows: CD45 = YKIX716.13, CD18 = YFC118.3 (human CD18), CD4 = YKIX302.9, CD8 = YCATE55.9, CD5 = YKIX322.3, CD21 = CA2.1D6, CD22 = RFB4 (human CD22, purchased from AbCam, Cambridge, MA), CD3 = CA17.2A12, CD14 = UCHM (human, used in panel 1) and CD14 = TUK4 (human, used in panel 2), class II MHC = YKIX334.2, CD34 = 1H6, CD25 = P2A10 (purchased from eBiosciences, San Diego, CA).

Clonality was confirmed in all cases using a PCR-based assay termed the PCR for antigen receptor rearrangements (PARR) assay [28,29], which detects clonal immunoglobulin gene rearrangements based on size and is similar to the method used in people [30].

CLL cases were selected at random from the CSU-CI database initially, with additional Boxers sequenced after discovering preferential IGHV gene usage in this breed.

### Case selection for large B-cell lymphoma and control dog cohorts

Cases with large B-cell lymphoma were identified among lymph node aspirate samples submitted to the CSU-CI laboratory for immunophenotyping. To meet the criteria for large B-cell lymphoma, >60% of the large cells in the sample expressed CD21 [31], and the median FS of the CD21+ lymphocytes was >450 U. Histopathology was not performed, so further subtyping could not be determined.

Control dogs without evidence of lymphoproliferative disease were identified at necropsy or biopsy and lymph node sections were collected.

### Characterization of the canine IGHV locus

**Fig 1:**
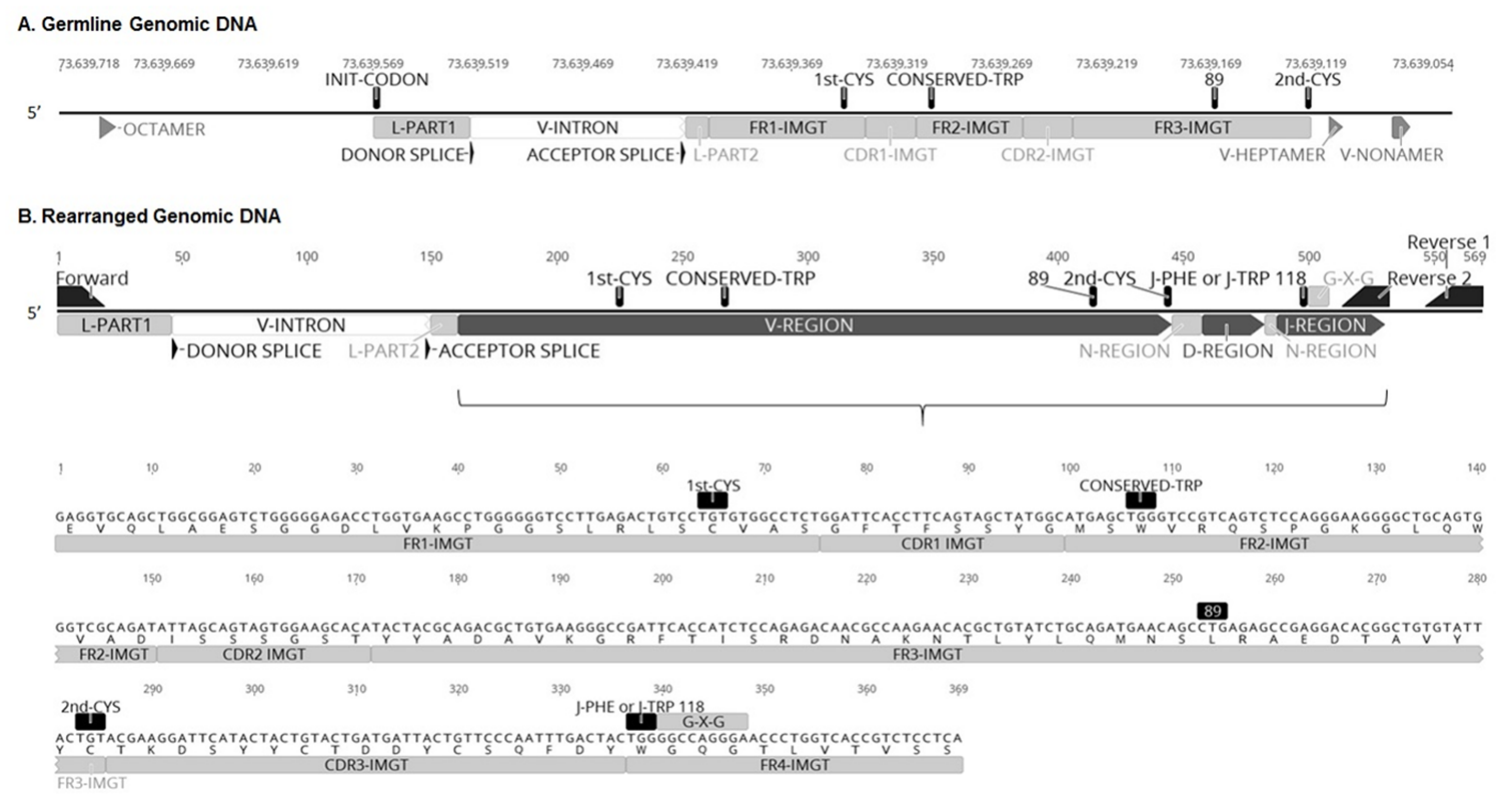
Dog IG V-REGION depictions in germline genomic DNA (A) and rearranged genomic DNA (B). (A) The V-DOMAIN of canine IGHV3-38 is shown as an example. IMGT standardized labels are shown, including framework regions (FR), complementarity determining regions (CDR), and the four conserved positions: 23 (1st-CYS), 41 (CONSERVED-TRP), 89 (hydrophobic) and 104 (2nd-CYS). Other labels include: the OCTAMER in the 5’UTR of the V-GENE; INIT-CODON, initiation codon (ATG sequence); L-PART1, first exon of the leader sequence; DONOR and ACCEPTOR SPLICE sites flanking the INTRON; L-PART2, second part of the leader sequence; V-HEPTAMER and V-NONAMER, recombination sites. (B) An IGHV-D-J rearrangement from a CLL case is shown as an example. The location of the PCR sequencing primers are shown, including the forward primer (used for both amplification protocols) and the reverse primers for protocol 1 (reverse 1) and protocol 2 (reverse 2)(see Table 1). Below, the genomic sequence and amino acid translation are shown for a portion of the rearrangement. The conserved position, 118 (J-PHE or J-TRP), and G-X-G motif of the J-REGION are shown.

In this study, we annotated the 80 germline genomic IGHV genes previously identified [18] using the guidelines established by the IMGT information system (http://imgt.cines.fr, [19–21]). A subset of these annotations are depicted in Fig 1. Productive rearrangements used for analysis had these conserved IMGT AA and motifs, and an open reading frame absent of stop codons or frameshift mutations.

Additionally, we identified new IGHV and IGHJ genes compared to previous annotations [18,22]. Contiguous germline DNA sequence from dog chromosome 8 (NCBI Reference Sequence: NW_003726071.1) encoding all previously identified IGHV genes through the immunoglobulin heavy constant mu (IGHM) gene was analyzed using the Geneious ‘Dotplot’ function with consensus IGHV and IGHJ gene probes generated via multiple sequence alignments (http://www.geneious.com, version 5.5.8 [32]). All identified genes were compared to those reported previously [18,22]. Newly identified genes were confirmed using BLAST search of dog genomic sequences (available at ncbi.nlm.nih.gov/projects/mapview, [33]) and/or dog BLAT search (available at genome.ucsc.edu, [34]), using the CanFam3.1 assembly (GenBank Assembly ID GCA_000002285.2).

### Sequencing of canine IGHV genes

For CLL cases, genomic DNA was extracted from 200 μL peripheral whole blood using the QIAamp DNA mini kit (Qiagen, Germantown, MD). DNA was amplified by PCR with a consensus IGHV3 subgroup-specific forward primer binding the leader exon (L-PART1) and either a pool of reverse primers binding the IGHJ intron regions (protocol 1) or the IGHJ coding regions (protocol 2) (Table 2; Fig 1). Earlier cases were sequenced using IGHJ intron primers, but these primers did not amplify rearrangements as effectively as IGHJ coding region primers, so the majority of cases were sequenced with protocol 2. While either leader primers or FR1 region primers may be used to amplify the IGHV region, leader primers were selected because they allow analysis of the whole IGHV gene, while FR1 primers exclude the 5’ portion [10]. Amplified products were purified using the DNA Clean & Concentrator-5 kit (Zymo Research Corp, Irvine, CA), ligated into a pDrive T-vector (Qiagen, Germantown, MD) and transformed into competent TG1 bacteria (Zymo Research Corp, Irvine, CA). Inserts were directly sequenced by Sanger sequencing from multiple independent clones (8–16 total). Tumor-associated IGHV-D-J rearrangements were identified as predominant repeated sequences with identical CDR3 sequences and a nucleotide length equal to the fragment size detected in the PARR assay. The cloning and sequencing protocol was identical for large B-cell lymphoma cases, except that DNA was isolated from fresh lymph node aspirates.

**Table 2:** PCR amplification primer sequences and cycling conditions for IGHV sequencing.

Primer sequence	Primer location	Cycling conditions
Protocol 1		
*Forward*: ATGGAGTCTGTGCTCGGCT	L-PART1	Denaturation step of 15 minutes at 95°C, followed by 10 cycles of 94°C for 30 seconds, 64°C for 30 seconds with the temperature decreasing 0.5°C every cycle, and 72°C for 1.5 minutes each, followed by 30 cycles of 94°C for 30 seconds, 59°C for 30 seconds and 72°C for 1.5 minutes each, and final extension step of 7 minutes at 72°C.
*Reverse primer pool*^a^: TGGATCTCAGGCTAACGGA CGGAAAGACAGACACAGGTAG CCCAGGAGTCTCTGGAAATTG CCCAGAGAGAAAGAGAAGAGAAG ATCTCTGCCTCCACTTCCT CCCAGAGAAAGGAGCAGAAA	IGHJ1-INTRON IGHJ2-INTRON IGHJ3-INTRON IGHJ4-INTRON IGHJ5-INTRON IGHJ6-INTRON	
Protocol 2		
*Forward*: ATGGAGTCTGTGCTCGGCT	L-PART1	Denaturation step of 5 minutes at 95°C, followed by 40 cycles of 94°C for 30 seconds, 60°C for 1 minute and 72°C for 1 minute each, and final extension step of 10 minutes at 72°C.
*Reverse primer pool*: ACCTGAGGAGACGGTGACC TGAGGACACGAAGAGTGAGG	J-REGION IGHJ2, IGHJ4 J-REGION IGHJ6	

^a^Protocol 1 reverse primer pool: amplification was first attempted with a pool of IGHJ2, IGHJ4, and IGHJ6 primers. If the clone was not identified, amplification was attempted in a second wave with a pool of IGHJ1, IGHJ3, and IGHJ5 primers. These primer sequences are located in the introns downstream of the IGHJ genes (see Fig 1B).

L-PART1: first exon, encoding the first part of the leader sequence in an IGHV gene; J-REGION: coding region of the IGHJ genes (see Fig 1B).

For control dogs without lymphoproliferative disease, more IGHV-D-J rearrangements were sampled per dog, since there was not a clonal B-cell population present. For each case, the PCR and cloning conditions were the same, except that DNA was extracted from a section of fresh whole lymph node and 100 independent clones were selected for sequencing, rather than 8–16 clones.

### Alignment and determination of mutational status and CDR3 length

Patient sequence from the first nucleotide of FR1 through the conserved 2nd-CYS in FR3 was queried against the CanFam3.1 assembly using NCBI BLAST (https://blast.ncbi.nlm.nih.gov). The most similar reference nucleotide sequence identified in this BLAST search was compared to our annotated library of germline reference IGHV genes (S1 Table) to determine IGHV gene usage in the patient. When more than one reference germline IGHV gene was identified as a possible match, the intron sequence was used to confirm the patient IGHV gene identity.

Mutational status was determined using guidelines adapted from human medicine [10]. The percentage of identity was calculated based on the number of nucleotide differences between the patient sequence and reference sequence in the V-REGION. Percent identity was calculated from the first nucleotide of FR1 to both the 2nd-CYS [35] and to codon 105 [10], but this boundary difference only changed the mutational status for one of the 389 sequences analyzed in this study. The sequence affected was a sequence from a control dog and did not have a statistical impact, so all sequences presented here were analyzed one way, to the 2nd-CYS. The following formula was used: IGHV identity (%) = 100 –(mutations/aligned IGHV region length×100), with an insertion or deletion of multiple nucleotides counted as one mutation. Cases were classified as unmutated when the percent identity was >98% and mutated when percent identity was <98%, according to the convention used for human CLL [10,36]. Mutated CLL cases were further categorized into those with percent identity between 96%-97.9% and those with <96% sequence homology [37,38]. The CDR3 length was identified by determining the number of codons from the first codon following 2nd-CYS to the last codon preceding TRP-118 [21]. Additionally, a subset of IGHV-D-J rearrangements were analyzed using the NCBI IgBLAST web-based program (http://www.ncbi.nlm.nih.gov/igblast/, [39]) and IMGT/V-QUEST (http://www.imgt.org/IMGT_vquest/vquest, version 3.4.8 [35,40]), to confirm that the same results were obtained by other analysis methods. Custom reference databases were used in IgBLAST by uploading the canine germline IGHV, IGHD and IGHJ gene libraries.

### Statistical analysis

A chi-square test and Fisher exact tests were performed to determine the statistical significance of differences in IGHV gene repertoire and mutational status.

## Results

### Study population

A total of 55 patients with CLL were included in the study, including 29 females and 26 males. The median age at the time of sample collection was 10.5 years (range, 4.9–15.6 years; one unknown). Initially, CLL cases were selected at random, with no bias for breed. However, as it became apparent that Boxer dogs had a unique IGHV gene usage, additional Boxer dogs were sequenced. Therefore, of the 55 CLL patients sequenced, 19 dogs (35%) were Boxers. Twenty-one breeds were represented in the non-Boxer group (Table 3). The peripheral B-cell count ranged from 7,300–816,600 cells/μL. Except for four more historic cases, all of the cases had a B-cell:neutrophil size ratio <0.55, which is the size cut-off used by Bromberek et al. [13] to define CLL cases. Four cases from 2011–2012 had a size ratio between 0.55–0.60, but were included because subjectively CD21+ lymphocytes appeared small by flow cytometry. Two of these four cases had a cytology report describing the lymphocytes as small and mature and suspicious for CLL, and two cases did not have cytology review.

**Table 3:** Breed, mutational status, and IGHV gene rearrangements in 55 canine patients with chronic lymphocytic leukemia.

Case No.	Breed^a^	Identity (%)	Mutational status^b^	IGHV	IGHJ	CDR3 length^c^
1	SHI	91.7	Mutated	IGHV3-75	IGHJ6	17
2	CDT	88.9	Mutated	IGHV3-47	IGHJ4	16
3	MIX	92.4	Mutated	IGHV3-47	IGHJ6	13
4	COC	93.1	Mutated	IGHV3-47	IGHJ4	22
5	RAT	87.8	Mutated	IGHV3-41	IGHJ4	11
6	BDC	99.0	Unmutated	IGHV3-41	IGHJ4	16
7	BIC	99.0	Unmutated	IGHV3-41	IGHJ4	18
8	AIR	100.0	Unmutated	IGHV3-41	IGHJ4	14
9	MIX	89.2	Mutated	IGHV3-38	IGHJ2	16
10	CKP	89.9	Mutated	IGHV3-38	IGHJ4	14
11	SHI	91.3	Mutated	IGHV3-38	IGHJ6	16
12	MLT	93.1	Mutated	IGHV3-38	IGHJ6	17
13	JRT	93.8	Mutated	IGHV3-38	IGHJ4	14
14	BIC	95.8	Mutated	IGHV3-38	IGHJ4	12
15	BIC	95.8	Mutated	IGHV3-38	IGHJ4	17
16	CRN	96.5	Borderline	IGHV3-38	IGHJ4	13
17	MIX	97.6	Borderline	IGHV3-38	IGHJ2	12
18	CSH	97.6	Borderline	IGHV3-38	IGHJ4	23
19	LAB	100.0	Unmutated	IGHV3-38	IGHJ4	15
20	WET	100.0	Unmutated	IGHV3-38	IGHJ4	16
21	CCR	94.8	Mutated	IGHV3-35	IGHJ4	14
22	MIX	91.0	Mutated	IGHV3-19	IGHJ4	13
23	SHI	91.0	Mutated	IGHV3-19	IGHJ2	14
24	CRN	94.1	Mutated	IGHV3-19	IGHJ4	13
25	SHI	94.8	Mutated	IGHV3-19	IGHJ4	13
26	MIX	94.8	Mutated	IGHV3-19	IGHJ4	14
27	PIT	95.5	Mutated	IGHV3-19	IGHJ4	10
28	MIX	100.0	Unmutated	IGHV3-19	IGHJ4	14
29	MIX	97.9	Borderline	IGHV3-12	IGHJ2	15
30	POM	98.6	Unmutated	IGHV3-9	IGHJ4	17
31	MIX	99.0	Unmutated	IGHV3-9	IGHJ6	12
32	LBD	92.0	Mutated	IGHV3-5	IGHJ4	16
33	BOR	95.1	Mutated	IGHV3-5	IGHJ4	13
34	SHI	95.5	Mutated	IGHV3-5	IGHJ2	14
35	STS	98.6	Unmutated	IGHV3-5	IGHJ2	27
36	LAB	95.8	Mutated	IGHV3-2	IGHJ4	15
37	BOX	99.0	Unmutated	IGHV3-67	IGHJ4	11
38	BOX	99.7	Unmutated	IGHV3-67	IGHJ4	10
39	BOX	100.0	Unmutated	IGHV3-67	IGHJ4	10
40	BOX	96.9	Borderline	IGHV3-41	IGHJ4	14
41	BOX	97.9	Borderline	IGHV3-41	IGHJ2	17
42	BOX	98.3	Unmutated	IGHV3-41	IGHJ4	17
43	BOX	99.3	Unmutated	IGHV3-41	IGHJ6	18
44	BOX	99.7	Unmutated	IGHV3-41	IGHJ4	11
45	BOX	99.7	Unmutated	IGHV3-41	IGHJ4	11
46	BOX	99.7	Unmutated	IGHV3-41	IGHJ6	22
47	BOX	100.0	Unmutated	IGHV3-41	IGHJ4	12
48	BOX	100.0	Unmutated	IGHV3-41	IGHJ4	13
49	BOX	100.0	Unmutated	IGHV3-41	IGHJ4	16
50	BOX	100.0	Unmutated	IGHV3-41	IGHJ6	17
51	BOX	97.9	Borderline	IGHV3-38	IGHJ4	13
52	BOX	100.0	Unmutated	IGHV3-38	IGHJ4	10
53	BOX	100.0	Unmutated	IGHV3-38	IGHJ4	14
54	BOX	96.5	Borderline	IGHV3-19	IGHJ4	12
55	BOX	99.3	Unmutated	IGHV3-5	IGHJ4	10

^a^Breed abbreviations: SHI, Shih-Tzu; CDT, Coton de Tulear; MIX, Mixed Breed; COC, Cocker Spaniel; RAT, Rat Terrier; BDC, Bearded Collie; BIC, Bichon Frise; AIR, Airedale Terrier; CKP, Cockapoo; MLT, Maltese; JRT, Jack Russell Terrier; CRN, Cairn Terrier; CSH, Chihuahua, Shorthair; LAB, Labrador Retriever; WET, Soft Coated Wheaten Terrier; CCR, Chinese Crested; PIT, Pit Bull Terrier; POM, Pomeranian; LBD, Labradoodle; BOR, Border Collie; STS, Schnauzer; BOX, Boxer.

^b^Mutational status: mutated (<96% similarity); borderline (96–98% similarity); unmutated (>98% similarity).

^c^Heavy chain complementary-determining region 3 amino acid length.

Eleven Boxer dogs with large B-cell lymphoma were sequenced, including four females and seven males. The median age at the time of sample collection was 8.5 years (range, 7.1–13.1 years).

Six dogs without lymphoproliferative disease were sequenced, including three Boxer dogs and three non-Boxer dogs. The Boxers ranged in age from 6.0–8.4 years and included one female and two males. The non-Boxers included one Labrador Retriever, one mixed breed dog, and one Chihuahua. The non-Boxers ranged in age from 9.0–13.0 years and included two females and one male. Five of six animals were deceased at the time of lymph node collection. One animal was alive at sample collection, diagnosed with mast cell tumor disease, and had five months follow up since sample collection. Two dogs died of heart failure and the remaining four dogs had non-lymphoid neoplasms. All six dogs had polyclonal immunoglobulin and T-cell receptor rearrangements by PARR, providing additional support that the dogs did not have lymphoproliferative disease (results not shown).

### Newly identified IGHV and IGHJ genes

**Fig 2:**
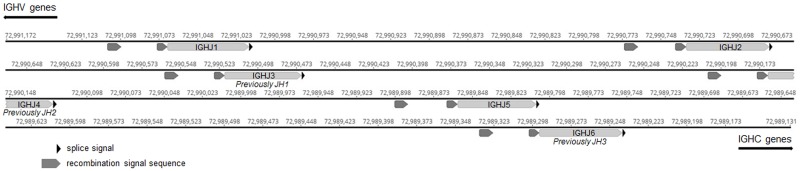
Dog IGHJ locus. The organization of the six canine IGHJ genes is shown, with the genomic locations on chromosome 8 identified (CanFam3.1, NCBI Accession NC_006590.3). Gene segments are shown as light grey bars, recombination signal sequences as dark grey bars, and splice signal sequences as black triangles. The three new IGHJ genes are IGHJ1, IGHJ2 and IGHJ5. The three IGHJ genes previously described [18] have been renamed from JH1, JH2 and JH3 to IGHJ3, IGHJ4 and IGHJ6, respectively, based on their genomic position. IGHC, immunoglobulin heavy constant genes.

A combination of BLAST/BLAT searching and Dotplot analyses were employed to identify seven new germline IGHV genes and three new germline IGHJ genes compared to those originally described by Bao et al. [18], bringing the totals to 87 IGHV and 6 IGHJ genes. All seven newly identified IGHV genes belong to the predominant IGHV3 subgroup, and were named: IGHV3-76, IGHV3-71, IGHV3-47-1, IGHV3-21-1, IGHV3-4, IGHV3-NL1, and IGHV3-NL2 (S1 Table). Three of these seven new IGHV genes were also annotated recently by Martin et al [22]. Five of the new IGHV genes mapped to chromosome 8 and two (designated NL) mapped to an unplaced genomic scaffold. Only IGHV3-76 and IGHV3-NL1 were considered functional. These two genes had open reading frames and conserved IMGT motifs, including the conserved amino acids: 23 (1st-CYS), 41 (CONSERVED-TRP), 89 (hydrophobic) and 104 (2nd-CYS). Two IGHV genes previously described [18] were reordered to reflect the genomic location on chromosome 8: IGHV3-32 (previously VH1-6) and IGHV3-78 (previously VH1-49P). The truncated sequence of VH1-42 that was previously published [18] was expanded and the more complete gene was renamed IGHV3-40. The three new IGHJ genes were clustered with the previously reported IGHJ genes on a 1.8 kb span 3’ to the IGHV region, and the IGHJ numbering system was revised from that which is already published [18], to IGHJ1-IGHJ6, 5’ to 3’ (Fig 2). This new annotation of the IGHJ genes is consistent with that described by Martin et al [22].

### CLL IGHV repertoire and mutational status

**Fig 3:**
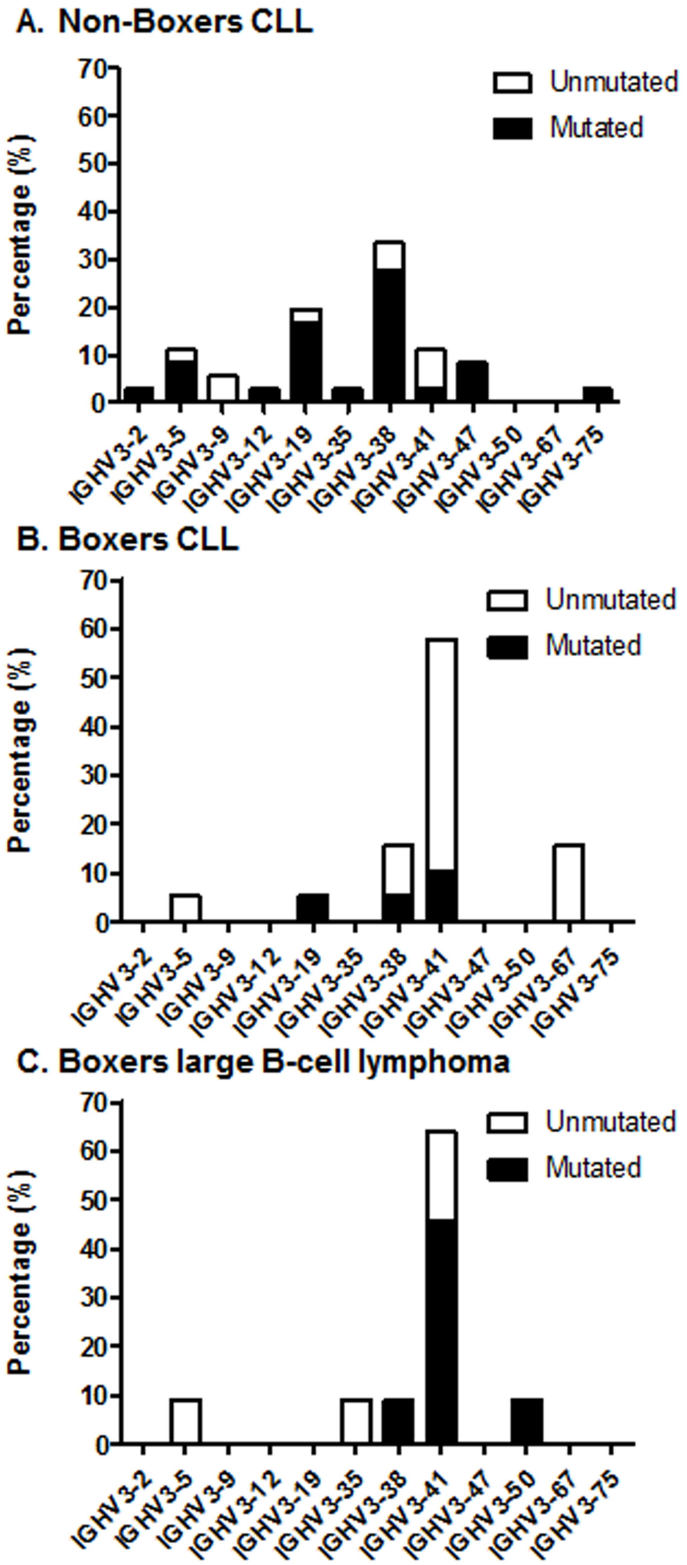
Distribution of IGHV gene usage and mutational status in (A) non-Boxer dogs with CLL (n = 36), (B) Boxer dogs with CLL (n = 19), and (C) Boxer dogs with large B-cell lymphoma (n = 11). IGHV gene usage is reported as the percentage of patients using an IGHV gene within that cohort. There were significant differences in the IGHV gene usage between non-Boxers and Boxers with CLL for IGHV3-67 (p = 0.037) and IGHV3-41 (p<0.001). There were significantly more unmutated cases in the Boxer CLL cohort, compared to non-Boxers with CLL (p<0.001) and Boxers with large B-cell lymphoma (p = 0.026).

IGHV-D-J rearrangements from 55 CLL patients were examined (Table 3). Eleven IGHV genes were represented in the CLL patient cohort. In non-Boxers, IGHV3-38 (33.3%) and IGHV3-19 (19.4%) were the most commonly used IGHV genes, followed by IGHV3-41 (11.1%) and IGHV3-5 (11.1%) (Fig 3A). In Boxers with CLL, IGHV3-41 (57.9%) was most commonly used, followed by IGHV3-38 (15.8%) and IGHV3-67 (15.8%) (Fig 3B). IGHV gene usage was significantly different between non-Boxers and Boxers for IGHV3-67 (p = 0.037) and IGHV3-41 (p<0.001).

Among non-Boxers with CLL, 9/36 (25%) cases were classified as unmutated and 27/36 (75%) cases were mutated. Among Boxers with CLL, 15/19 (79%) cases were unmutated, while 4/19 (21%) cases were mutated. The majority of mutated non-Boxer CLL cases had a percent identity <96% (24/36 (66.7%) cases), and few cases with a 96%-97.9% identity (3/36 (8.3%) cases). All four mutated Boxer CLL cases had a percent identity between 96%-97.9%, with none of the cases having a percent identity <96%. The frequency of unmutated cases was significantly higher in Boxers with CLL compared to other breeds (p<0.001).

The IGHJ gene repertoire was not significantly different between non-Boxer and Boxer CLL patients. Across the cohort of 55 patients, 3 IGHJ genes rearranged. IGHJ4 (72.7%) was the most frequently used IGHJ gene, followed by IGHJ6 (14.5%) and IGHJ2 (12.7%). The heavy chain CDR3 mean length was 14.6 AA (range, 10–27 AA) across all CLL cases. There was not a significant difference in CDR3 length between breed groups or between mutated and unmutated cases.

These results indicate that Boxers with CLL have preferential rearrangement of IGHV3-41 and that the majority of cases are unmutated, regardless of IGHV gene usage.

### Large B-cell lymphoma IGHV repertoire and mutational status

To determine whether Boxers with other forms of lymphoproliferative disease have preferential rearrangement of IGHV3-41 or unmutated IGHV genes, IGHV-D-J rearrangements from 11 Boxers with large B-cell lymphoma were examined. IGHV3-41 (63.6%) was most commonly used, at a frequency similar to that seen in the Boxer CLL cohort (Fig 3C). 4/11 (36.4%) cases were classified as unmutated, and 7/11 (63.6%) cases were mutated. Among mutated cases, one case had a percent identity between 96%-97.9%, and remaining mutated cases had <96% homology to germline. The IGHJ gene repertoire was similar to CLL, with IGHJ4 (72.7%) most frequently used, followed by IGHJ6 (18.2%) and IGHJ2 (9.1%). The CDR3 mean length was 13.8 AA (range, 10–20 AA). These results indicate that Boxers with large B-cell lymphoma preferentially use IGHV3-41, as seen in Boxers with CLL, but in the majority of cases the IGHV genes are mutated rather than unmutated.

### Normal canine IGHV repertoire and mutational status

**Fig 4:**
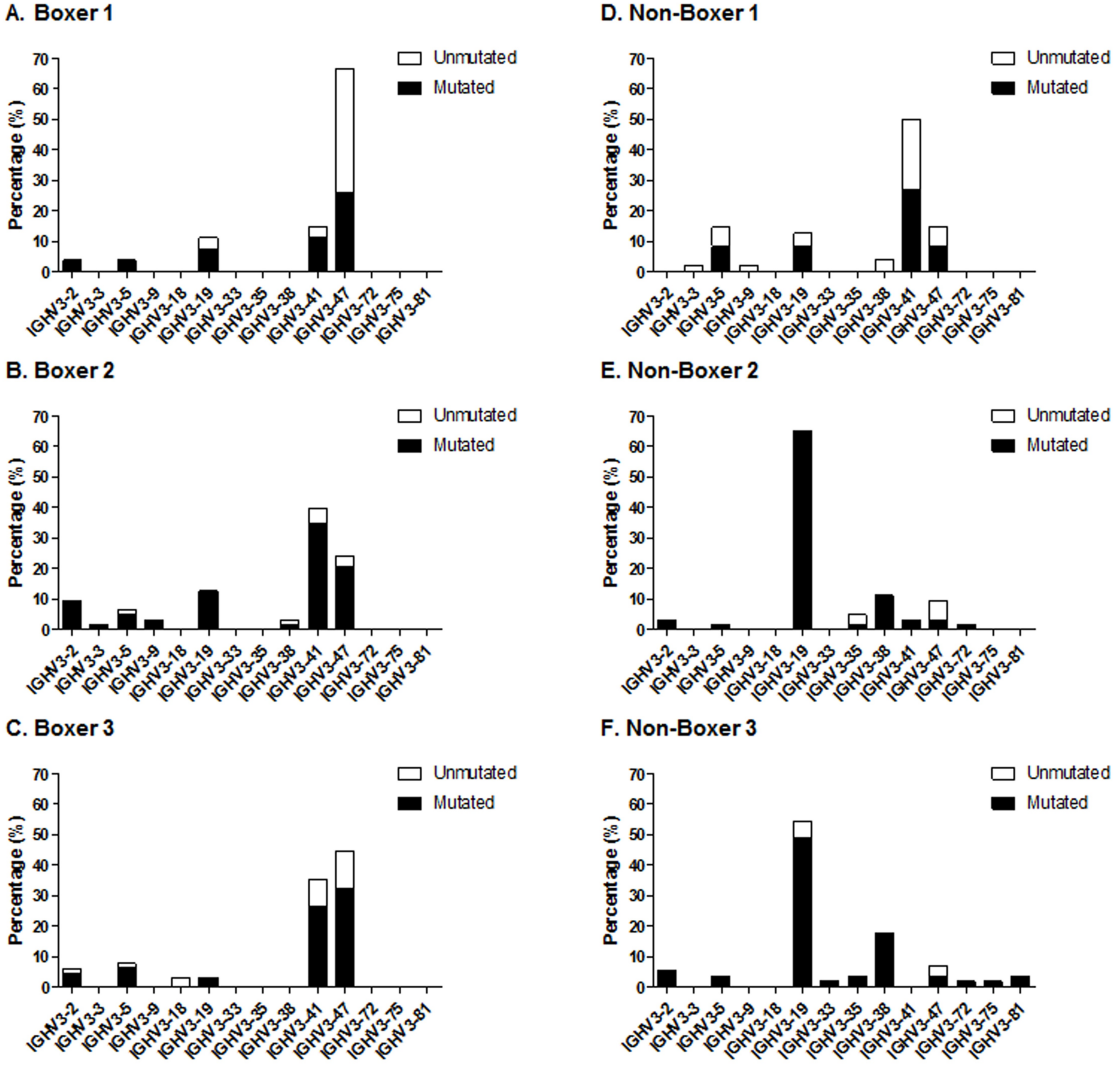
Distribution of IGHV gene usage and mutational status in 3 Boxer dogs (A-C) and 3 non-Boxer dogs (D-F) without lymphoproliferative disease. IGHV gene segment usage is reported as the percentage of unique rearrangements using an IGHV gene within a single dog’s repertoire. There were significant differences in the IGHV gene usage between Boxers and non-Boxers for IGHV3-47, IGHV3-41, IGHV3-38, and IGHV3-19 (p<0.001 for all genes). There was no significant difference in the mutational status between Boxers and non-Boxers. The number of unique rearrangements examined for each case included: Boxer 1, n = 27; Boxer 2, n = 63; Boxer 3, n = 65; non-Boxer 1 (Labrador Retriever), n = 48; non-Boxer 2 (Mixed Breed), n = 63; non-Boxer 3 (Chihuahua), n = 57.

IGHV-D-J rearrangements from six control dogs without lymphoproliferative disease were examined, including three non-Boxers and three Boxers. The number of unique productive clones obtained for each animal ranged from 27 to 65 (Fig 4). Across the six dogs, which included 323 unique IGHV-D-J rearrangements, the most commonly used IGHV genes were IGHV3-19 (28.2%), IGHV3-47 (24.5%), and IGHV3-41 (24.1%), followed by IGHV3-38 (6.5%), IGHV3-5 (6.2%) and IGHV3-2 (5.0%). There were significant differences in IGHV gene usage between individual animals, and between breeds. Boxers (Fig 4A–4C) used IGHV3-41 (p<0.001) and IGHV3-47 (p<0.001) significantly more than non-Boxers, and non-Boxers (Fig 4D–4F) used IGHV3-19 (p<0.001) and IGHV3-38 (p<0.001) significantly more than Boxers. There was no significant difference in mutational status between Boxers and non-Boxers. These results indicate that Boxers with normal B-cells preferentially use IGHV3-41 and IGHV3-47 compared to other breeds, and the majority of rearrangements are mutated.

## Discussion

In this study, we investigated IGHV gene usage and mutational status in canine patients with CLL. Among non-Boxer patients with CLL, we found that the majority of cases (75%) were mutated, using the homology cut-off value of 98% as established in human CLL. The ratio of mutated to unmutated cases was slightly higher than seen in the human population [6,41]. Boxers were analyzed separately as they were preferentially sequenced over other breeds due to their skewed use of unmutated IGHV genes. Among Boxers with CLL, 79% of cases were unmutated and all of the mutated cases had a percent identity between 96%-97.9%, demonstrating that none of the Boxer CLL cases sequenced were highly mutated. These data suggested that the Boxer breed may be a useful model for unmutated CLL. However, the reference dog genome sequence was obtained by sequencing a Boxer [42]; therefore, we were concerned that the high homology between the Boxer CLL cases and reference genome may be due to breed bias. To address this question, we sequenced IGHV genes from additional Boxer dogs with large B-cell lymphoma and without lymphoproliferative disease and found that these dogs used predominantly mutated IGHV genes. The majority of IGHV genes from Boxers with large B-cell lymphoma (64%) were mutated. There was no significant difference in the number of unmutated IGHV gene rearrangements in normal Boxers compared to other breeds, suggesting use of unmutated IGHV genes in Boxers is specific to CLL.

We examined gene usage in CLL patients as well as the normal IGHV gene repertoire in six control dogs. IGHV3-38 and IGHV3-19 were most commonly used in non-Boxers with CLL and in the normal gene repertoire of two of the three non-Boxer control dogs. One of the three control non-Boxers had a gene repertoire more similar to the Boxers. Bao et al. [18] demonstrated a bias for IGHV3-38 and IGHV3-19 amongst three healthy dogs, and IGHV3-38 was preferentially rearranged in canine cases with diffuse large B-cell lymphoma [25,26]. Boxers with CLL, large B-cell lymphoma, and without lymphoproliferative disease preferentially used IGHV3-41, indicating IGHV3-41 gene usage is high in Boxers, regardless of disease status. IGHV3-47 was commonly used in all three control Boxers without lymphoproliferative disease, but was not used in Boxers with CLL or large B-cell lymphoma. IGHV3-67 was one of the more commonly used IGHV genes in Boxers with CLL, but was not used in any of the non-Boxers with CLL, Boxers with large B-cell lymphoma, or normal B-cell repertoires examined. This suggests IGHV3-67 may be preferentially used in Boxers with CLL, but the number of cases is small and additional cases are needed to verify this finding. A limitation of this study is that the leader primer used for sequencing is specific to the IGHV3 subgroup, which likely had a small effect on the normal repertoire of control dogs. The IGHV3 subgroup is the dominant subgroup in multiple studies [18,23,24], accounting for 90% or more of rearranged genes in one study [23], and we were able to identify the clone in 92% of CLL cases we attempted to sequence with an IGHV3-specific primer.

This study contributes to our understanding of the canine immunoglobulin heavy chain variable region. Seven new IGHV genes and three new IGHJ genes were identified compared to the original annotation from Bao et al [18]. Some of the additions may be attributed to different reference genome builds. Martin et al. recently described three of these new IGHV genes and the new IGHJ genes [22]. Slight differences in our annotations may be due to different consensuses used to search for new IGHV genes. Additionally, all of the IGHV genes were assessed for functionality and annotated using the IMGT guidelines. All productive rearrangements used for analysis had the four previously described conserved AA and the conserved Gly-X-Gly motif following codon 118. Eight of 389 sequences examined in this study had a residue other than the highly conserved PHE or TRP at codon 118, but were considered adequate for interpretation because the Gly-X-Gly was conserved [11].

A challenge of mutational analysis in dogs is the lack of knowledge about polymorphisms in IGHV genes across dog breeds and individuals. Ideally, canine CLL sequences would be compared to reference genome sequences from the same breed, but those are not available at this time. Additionally, the homology cut-off of 98% which distinguishes clinically distinct subsets of human CLL patients [5,6,43] may need to be adjusted to distinguish subsets of canine patients with different prognoses. The next phase of this study is to perform a large-scale outcome study in dogs with CLL to correlate clinical outcome with mutational status. An additional challenge is the lack of antibodies available to differentiate CLL from other canine B-cell neoplasms. The case criteria used in this study define an entity in dogs that most closely resembles CLL in people in its clinical presentation [13]. However, this entity may represent a different small B-cell neoplasm with lymphocytosis, such as leukemic mantle cell lymphoma [44], or group of small B-cell neoplasms. We consider leukemic mantle cell lymphoma a less likely differential because this histologic subtype appears quite rare in dogs [45–47]. Future steps include obtaining histopathology and gene expression profiling to correlate canine findings with that seen in human CLL.

These results contribute to our understanding of the canine immunoglobulin genes and are the first to examine mutational status in a canine population with CLL. This study identified Boxers with CLL as having predominantly unmutated IGHV gene rearrangements and no highly mutated rearrangements. This suggests that this breed may be a valuable model to study CLL associated with unmutated IGHV genes.

## Supporting information

S1 Table Canine IGHV genes, including the IMGT name, previous name, genomic location, and functionality information. (DOCX)
