# Supplementary material for: Preferential use of unmutated immunoglobulin heavy variable region genes in Boxer dogs with chronic lymphocytic leukemia
Source: PLoS One. 2018 Jan 31;13(1):e0191205. doi: 10.1371/journal.pone.0191205 (PMC5791963; doi:10.1371/journal.pone.0191205)
Supplement: S1 Table — (DOCX) [file pone.0191205.s001.docx]

| **IMGT gene name and allele^a^** | **IMGT gene order** | **Previous gene name^b^** | **Functionality^c^** | **INIT-CODON position^d^** | **Open reading frame** | **OCTAMER sequence** | **INIT-CODON sequence** | **Splice site sequence** | **V-HEPTAMER sequence** | **V-NONAMER sequence** | **Slightly altered gene^e^** | **Highly altered gene^f^** | **New IGHV gene^g^** |
| --- | --- | --- | --- | --- | --- | --- | --- | --- | --- | --- | --- | --- | --- |
| IGHV3-83*01 | 1 | VH1-01P | P | 74271225 | no | ATGCAAAT | ATG | GT/AG | CACAGCG | ATAGAAACC | • |  |  |
| **IGHV3-82*01** | 2 | VH1-02 | F | 74248215 | yes | ATGCAAAT | ATG | GT/AG | CACAGTG | ACACAAACC |  |  |  |
| **IGHV3-81*01** | 3 | VH1-03 | F | 74242927 | yes | ATGCAAAT | ATG | GT/AG | CACAGTG | ACACAAACC |  |  |  |
| **IGHV3-80*01** | 4 | VH1-04 | F | 74224404 | yes | ATGCAAAT | ATG | GT/AG | CACAGTG | ACACAAACC |  |  |  |
| IGHV3-79*01 | 5 | VH1-05P | P | 74221247^*^ | no | n/a | n/a | n/a | CACTGTG | ACACAAACC |  | • |  |
| IGHV3-78*01 | 6 | VH1-49P | P | 74212847 | no | ATGCAAAT | ATG | GT/AG | CACAGAG | ACAAACCTC | • |  |  |
| IGHV3-77*01 | 7 | VH1-07P | P | 74196987^*^ | no | n/a | n/a | TT/AG | CACAGTG | ACACAAACC |  | • |  |
| **IGHV3-76*01** | 8 | n/a | F | 74166350 | yes | ATGCAAAT | ATG | GT/AG | CACCGTG | ATACACACC |  |  | • |
| **IGHV3-75*01** | 9 | VH1-08 | F | 74144648 | yes | ATGCAAAG | ATG | GT/AG | CACAGTG | ATAGAAACC |  |  |  |
| IGHV3-74*01 | 10 | VH1-09P | P | 74133429 | no | AAGCAAAT | ATG | GT/AG | CACAATG | ACACAAACC | • |  |  |
| IGHV3-73*01 | 11 | VH1-10P | P | 74108799 | no | ATATAAAT | ATG | GT/AG | CACAGTG | ACACAAACA | • |  |  |
| IGHV3-72*01 | 12 | VH1-11P | P | 74102005 | no | ATGCAAAT | ATG | GT/AG | CACAGTG | ACACAAACC | • |  |  |
| IGHV3-71*01 | 13 | n/a | P | 74094207 | no | ATGCAAAT | ATG | GT/AG | CACATTG | AGAAATCTC |  | • | • |
| **IGHV3-70*01** | 14 | VH1-12 | F | 74080028 | yes | ATGCAAAT | ATG | GT/AG | CACAGTG | ACACAAACT |  |  |  |
| **IGHV3-69*01** | 15 | VH1-13 | F | 74068281 | yes | ATGCAAAT | ATG | GT/AG | CACAGTG | ACACAAACC |  |  |  |
| IGHV3-68*01 | 16 | VH1-14P | P | 74057772 | no | ATGCAAAT | ATG | GT/AG | CACAGTG | ACACAAACC | • |  |  |
| **IGHV3-67*01** | 17 | VH1-15 | F | 74052503 | yes | ATGCAAAT | ATG | GT/AG | CACAGTG | ACACAAACC |  |  |  |
| IGHV3-66*01 | 18 | VH1-16P | P | 74045423 | no | ATACAAAT | CTG | GT/AG | CACAGTG | ACACAAACC | • |  |  |
| IGHV3-65*01 | 19 | VH1-17P | P | 74036077 | no | ATGAAAAT | ATG | GT/AG | CACTGTG | ACACAAACC |  | • |  |
| IGHV3-64*01 | 20 | VH1-18P | P | 74028070 | no | ATGCAAAT | ATG | GT/AG | CACAGTG | ACACAAACC |  | • |  |
| IGHV3-63*01 | 21 | VH1-19P | P | 74012210^*^ | no | n/a | n/a | TT/AG | CACAGTG | ACACAAACC |  | • |  |
| IGHV3-62*01 | 22 | VH1-20P | P | 73992957 | no | ATACAAAA | ATG | GT/TG | CACAGTG | ACACAAACA | • |  |  |
| **IGHV3-61*01** | 23 | VH1-21 | F | 73981005 | yes | ATGCAAAT | ATG | GT/TG | CACATTG | ACACAAACC |  |  |  |
| IGHV3-60*01 | 24 | VH1-22P | P | 73974531 | no | ATGCAAAT | ATG | AT/AG | CACATTG | ACAGAAATC |  | • |  |
| IGHV3-59*01 | 25 | VH1-23P | P | 73968132 | no | ATGCAAAT | ATG | GT/AG | CACAGTG | ACACAAACT | • |  |  |
| **IGHV3-58*01** | 26 | VH1-24 | F | 73956330 | yes | ATGCAAAT | ATG | GT/AG | CACAGTG | ACAGAAACC |  |  |  |
| IGHV3-57*01 | 27 | VH1-25P | P | 73942869^*^ | no | n/a | n/a | GT/AG | CAGAGTG | ACACAAACC |  | • |  |
| IGHV3-56*01 | 28 | VH1-26P | P | 73939635 | no | ATGCAAAT | ATG | GT/AG | CACAGTG | ACACAAACC |  | • |  |
| IGHV3-55*01 | 29 | VH1-27P | P | 73906008 | no | ATGCAAAT | ATG | GT/AG | CACAGTG | ACACAAAGC | • |  |  |
| **IGHV3-54*01** | 30 | VH1-28 | F | 73886557 | yes | ATGCAAAT | ATG | GT/AG | CACAATG | ACACAAACC |  |  |  |
| IGHV3-53*01 | 31 | VH1-29P | P | 73865820^*^ | no | n/a | n/a | GT/AG | CACAGTG | ACACAAACC |  | • |  |
| IGHV3-52*01 | 32 | VH1-30P | P | 73851259 | no | ATACAAAT | ATG | GT/AG | CACAGTG | ACACAAACC | • |  |  |
| IGHV3-51*01 | 33 | VH1-31P | P | 73845797 | no | ATGCAAAT | ATG | GT/AG | CACAGTG | ACACAAACC |  | • |  |
| **IGHV3-50*01** | 34 | VH1-32 | F | 73838094 | yes | ATGCAAAT | ATG | GT/AG | CACAGTG | ACACAAACC |  |  |  |
| IGHV3-49*01 | 35 | VH1-33P | P | 73829948 | no | n/a | ATG | GT/AG | CACAGAG | ACACAAACC |  | • |  |
| IGHV3-48*01 | 36 | VH1-34P | P | 73814217^*^ | no | n/a | n/a | TT/AG | CACAGTG | ACACAAACC |  | • |  |
| IGHV3-47-1*01 | 37 | n/a | P | 73774647 | no | ATGCAAAT | ATG | GT/AG | CACCGTG | ATACACACC | • |  | • |
| **IGHV3-47*01** | 38 | VH1-35 | F | 73751519 | yes | ATGCAAAT | ATG | GT/AG | CACAGTG | ACACAAACC |  |  |  |
| **IGHV3-46*01** | 39 | VH1-36 | F | 73744495 | yes | ATGCAAAT | ATG | GT/AG | CACAGTC | ACACAAACC |  |  |  |
| IGHV3-45*01 | 40 | VH1-37P | P | 73734991^*^ | no | n/a | n/a | GT/AG | TGCAGTG | ACAGAAACC |  | • |  |
| IGHV3-44*01 | 41 | VH1-38P | P | 73729795 | yes | ATGCAAAT | ATG | GA/AG | CACAGTG | ACACAAACC | • |  |  |
| IGHV3-43*01 | 42 | VH1-39P | P | 73713601 | no | ATGCAAAT | ATG | GT/AG | CACAATG | ACACAAACC | • |  |  |
| IGHV3-42*01 | 43 | VH1-40P | P | 73702821 | no | ATGCAAAT | ATG | GT/AG | CACAGAG | ACACAAACC |  | • |  |
| **IGHV3-41*01** | 44 | VH1-41 | F | 73686189 | yes | ATGCAAAT | ATG | GT/AG | CACAGTG | ACACAAACC |  |  |  |
| IGHV3-40*01 | 45 | VH1-42P | P | 73663825 | no | ATGCAAAT | ATG | GT/AG | CACAGTG | ACACAAACG | • |  |  |
| **IGHV3-39*01** | 46 | VH1-43 | F | 73655490 | yes | ATGCAAAT | ATG | GT/AG | CACAGTG | ACACAAACC |  |  |  |
| **IGHV3-38*01** | 47 | VH1-44 | F | 73639568 | yes | ATGCAAAT | ATG | GT/AG | CACAGTG | ACACAAACC |  |  |  |
| **IGHV3-37*01** | 48 | VH1-45 | F | 73620033 | yes | ATGCAAAT | ATG | GT/AG | CACAGTC | ACACAAACC |  |  |  |
| IGHV3-36*01 | 49 | VH1-46P | P | 73600284 | no | ATGCAAAT | ATG | GT/AG | TACAGTT | ACACAAACC | • |  |  |
| **IGHV3-35*01** | 50 | VH1-47 | F | 73595945 | yes | ATGCAAAT | ATG | GT/AG | CACAGTG | ACACAAAAC |  |  |  |
| **IGHV3-33*01** | 52 | VH1-48 | F | 73580014 | yes | ATGCAAAT | ATG | GT/AA | CACAGTG | ACACAAACC |  |  |  |
| **IGHV3-32*01** | 53 | VH1-06 | F | 73576558 | yes | ATGCAAAT | ATG | GT/AG | TACAGTG | ACACAAAAT |  |  |  |
| IGHV3-31*01 | 54 | VH1-50P | P | 73565713 | yes | ATGCAAAT | ATG | GT/AG | CACAATG | ACACAATCC | • |  |  |
| **IGHV1-30*01** | 55 | VH2-51 | F | 73558100 | yes | ATGCAAAT | ATG | GT/AG | CACAGTG | TCAGAAACC |  |  |  |
| IGHV3-29*01 | 56 | VH1-52P | P | 73547836 | no | ATGCAAAT | ATG | GT/AG | CACAGTG | GCACAAACC | • |  |  |
| IGHV3-28*01 | 57 | VH1-53P | P | 73534024 | no | ATGCAAAT | ATG | GT/AG | CACAGTG | ACACAAACC | • |  |  |
| IGHV3-27*01 | 58 | VH1-54P | P | 73520719 | no | ATGCAAAT | ATG | GT/AG | CACAGTG | ACACAAACC | • |  |  |
| IGHV3-26*01 | 59 | VH1-55P | P | 73509474 | no | ATGCAAAT | ATG | GT/AG | CACAGTG | ACACAAACC | • |  |  |
| IGHV3-25*01 | 60 | VH1-56P | P | 73487223 | no | ATGCAAAC | ATG | GT/AG | CACAGTG | ATACAAACC | • |  |  |
| **IGHV3-24*01** | 61 | VH1-57 | F | 73478222 | yes | ATGCAAAT | ATG | GT/AG | CACAGTG | ACACTCACT |  |  |  |
| **IGHV3-23*01** | 62 | VH1-58 | F | 73466463 | yes | ATGCAAAT | ATG | GT/AG | CACAGTG | ACACAAACC |  |  |  |
| IGHV3-22*01 | 63 | VH1-59P | P | 73450839 | no | ATGCAAAT | ATG | GT/AG | CACAGTG | ACACAAACC | • |  |  |
| IGHV3-21-1*01 | 64 | n/a | P | 73442614^*^ | no | n/a | n/a | AG | CACATTG | ACAGAAGTC |  | • | • |
| IGHV3-21*01 | 65 | VH1-60P | P | 73430644 | no | ATGCAAAT | ATG | AT/AG | CACAATG | ACACAAACC | • |  |  |
| IGHV3-20*01 | 66 | VH1-61P | P | 73412008 | no | ACACAAAT | ATG | GT/AG | CAAAGTG | ACACAAACC |  | • |  |
| **IGHV3-19*01** | 67 | VH1-62 | F | 73387704 | yes | ATGCAAAT | ATG | GT/AG | CACAGTG | ACACAAACC |  |  |  |
| **IGHV3-18*01** | 68 | VH1-63 | F | 73365401 | yes | ATGCAAAT | ATG | GT/AG | CACAGTG | ACACAAAAC |  |  |  |
| IGHV1-17*01 | 69 | VH2-64P | P | 73327998 | no | n/a | ATG | GT/AG | GACAGTG | TCAGAAACC | • |  |  |
| **IGHV3-16*01** | 70 | VH1-65 | F | 73306390 | yes | ATGCAAAG | ATG | GT/AG | CACAGTG | ACACAAACC |  |  |  |
| IGHV1-15*01 | 71 | VH2-66P | P | 73295459 | no | ATGCAAAT | ATG | GT/AG | CACAGTG | TCAGAAACC | • |  |  |
| IGHV3-14*01 | 72 | VH1-67P | P | 73272544 | no | ATGCAAAT | ATG | GT/AG | CACAGTG | ACACAAACC | • |  |  |
| **IGHV3-13*01** | 73 | VH1-68 | F | 73267073 | yes | ATGCAAAT | ATG | GT/AG | CACAGTG | ACACAAACC |  |  |  |
| IGHV3-12*01 | 74 | VH1-69P | P | 73235778 | no | ATGCAAAT | ATG | GT/AG | CACAATG | ACACAAACC | • |  |  |
| IGHV3-11*01 | 75 | VH1-70P | P | 73216544 | no | ATTCAAAT | ATG | GT/AC | CAGAGAG | AGACACAAA | • |  |  |
| **IGHV3-10*01** | 76 | VH1-71 | F | 73195153 | yes | ATGCAAAT | ATG | GT/AG | CACCGTG | ACACAAACC |  |  |  |
| **IGHV3-9*01** | 77 | VH1-72 | F | 73185283 | yes | ATGCAAAT | ATG | GT/AG | CACAGTG | ACACAAACC |  |  |  |
| **IGHV3-8*01** | 78 | VH1-73 | F | 73159977 | yes | ATGCAAAT | ATG | GT/AG | CACAATG | ACACAAACC |  |  |  |
| **IGHV3-7*01** | 79 | VH1-74 | F | 73153457 | yes | ATGCAAAT | ATG | GT/AG | CACAGTG | ACACAAACC |  |  |  |
| **IGHV3-6*01** | 80 | VH1-75 | F | 73137027 | yes | ATGCAAAT | ATG | GT/AG | CACAGTG | ACACAAACC |  |  |  |
| IGHV3-5-1*01 | 81 | VH1-76P | P | 73123182 | no | ATGCAAAT | ATG | GT/AG | CACAATG | ACACAAACC |  | • |  |
| **IGHV3-5*01** | 82 | VH1-77 | F | 73109226 | yes | ATGCAAAT | ATG | GT/AC | CACCGTG | ACACAAACC |  |  |  |
| IGHV3-4*01 | 83 | n/a | P | 73068674 | no | TTGCAAAT | ATG | GT/AC | CACAGTG | ACACAAACC | • |  | • |
| **IGHV3-3*01** | 84 | VH1-78 | F | 73056536 | yes | ATGCAAAT | ATG | GT/AG | CACAGTG | ACACAAACT |  |  |  |
| **IGHV3-2*01** | 85 | VH1-79 | F | 73038393 | yes | ATGCAAAT | ATG | GT/AG | CACAGTA | ACACAAACC |  |  |  |
| **IGHV4-1*01** | 86 | VH3-80 | F | 73028843 | yes | ATGCAAAT | ATG | GT/AG | CACAGTG | ACACAAACC |  |  |  |
| **IGHV3-NL1*01** | 0 | n/a | F | n/a | yes | ATGCAAAT | ATG | GT/AG | CACAGTG | ACACAAACC |  |  | • |
| IGHV3-NL2*01 | 0 | n/a | P | n/a | no | ATACAAAG | ATG | AT/AG | CGCAGGG | ACGCAAACC |  | • | • |

^a^The IMGT name is provided for the canine IGHV genes (http://www.imgt.org, release 201715-4 (13 April 2017)).

^b^The previous IGHV gene name from Bao et al. [18] is listed.

^c^IGHV genes are designated as functional (F) or pseudogenes (P). The IMGT name is bolded for functional canine IGHV genes.

^d^The genomic location on chromosome 8 is identified for the start of the initiation codon, INIT-CODON (CanFam3.1, NCBI Accession NC_006590.3). Pseudogenes without an INIT-CODON are indicated with an asterisk, and the genomic location provided is the start of the V-HEPTAMER of the V-RS (recombination sequence).

The sequence for the following gene elements is provided: OCTAMER, INIT-CODON, ACCEPTOR-SPLICE AND DONOR-SPLICE sites, V-HEPTAMER and V-NONAMER. ‘n/a’ indicates that the gene element was not found.

^e^Pseudogenes that are slightly altered are identified with an ‘•.’ These are pseudogenes in the reference Boxer genome, but appear to have the potential to be functional if there were minor changes/polymorphisms in the sequence.

^f^Pseudogenes that are highly altered are identified, indicating these genes have major alterations and do not appear to have the potential to be functional.

^g^Genes indicated with an ‘•’ are newly identified IGHV genes, that were not previously reported in Bao et al. [18].
